# Diagnosis of canine B-cell chronic lymphoid leukemia with a CD21 negative phenotype using the LT21 clone CD21 antibody in flow cytometry: a case report

**DOI:** 10.1186/s12917-024-04335-x

**Published:** 2024-10-26

**Authors:** Eun Wha Choi, Yunho Jeong, Jin-Ok Ahn

**Affiliations:** 1 Department of Veterinary Clinical Pathology, College of Veterinary Medicine & Institute of Veterinary Science, Kangwon National University, 1 Kangwondaehak-gil, Chuncheon, Gangwon-do 24341 Republic of Korea; 2 Department of Veterinary Internal Medicine, College of Veterinary Medicine, Kangwon National University, 1 Kangwondaehak-gil, Chuncheon, Gangwon-do 24341 Republic of Korea

**Keywords:** B-cell chronic lymphoid leukemia, CD21, Immunophenotyping, Dog, Lymphoproliferative diseases

## Abstract

**Background:**

Chronic lymphoid leukemia (CLL) is a hematological disorder characterized by the clonal expansion of small mature lymphocytes that accumulate in the blood and bone marrow. CLL can arise from B-, T-, or natural killer cell clones. The cytological evaluation of blood smears is often the simplest and least invasive method for diagnosing lymphoid leukemia. Immunophenotyping is used to further subclassify the type of lymphoid leukemia.

**Case presentation:**

A 15-year-old, 4.4-kg spayed female Shih Tzu was presented to the veterinary medical teaching hospital of Kangwon National University. Despite having a normal appetite and activity level, cervical and inguinal lymph node enlargement was noted on physical examination. Complete blood count revealed severe leukocytosis, severe lymphocytosis, and monocytosis. Splenomegaly, hepatomegaly, and lymph node enlargement were detected on radiographic and ultrasonographic examination. Immunophenotyping was performed using peripheral blood mononuclear cells (PBMCs). The majority of lymphocytes exhibited the following profiles: CD3^−^CD79a^−^ (97.5%), CD4^−^CD8^−^ (98.6%), CD21^−^CD79a^−^ (98.4%), CD34^−^ (0.1%), CD45^+^ (99.6%), major histocompatibility complex class II^+^ (99.5%), and CD14^−^ (0.5%). Based on the immunophenotyping results, possible differentials considered included the following: the majority of lymphocytes may be natural killer (NK) cell clones, plasma cell clones, or show aberrant expression or loss of CD21 marker due to the neoplastic nature of the cells. Further flow cytometry was performed using antibodies against CD3, CD5, CD94, and granzyme B. The combined results indicated that the predominant lymphocyte subset in the PBMCs was CD3^−^CD5^−^CD21^−^CD94^−^granzyme B^−^. To confirm monoclonality and exclude the aberrant loss of CD markers, a polymerase chain reaction for antigen receptor rearrangement (PARR) assay was conducted. The PARR assay, using DNA from blood and lymph node samples, showed B-cell monoclonality. Immunocytochemistry using PBMCs showed that the plasma cell marker Multiple Myeloma Oncogene 1 (MUM1) was not expressed. Therefore, the diagnosis was confirmed to be B-cell CLL.

**Conclusion:**

Immunophenotyping can help subclassify the type of lymphoid leukemia; however, as tumor cells can show aberrant expression or loss of the CD21 marker, combining immunophenotyping with the PARR assay could yield a more accurate diagnosis.

**Supplementary Information:**

The online version contains supplementary material available at 10.1186/s12917-024-04335-x.

## Background

Chronic lymphoid leukemia (CLL) is a hematologic disorder characterized by the clonal expansion of small mature lymphocytes that accumulate in the blood and bone marrow [1, 2]. Lymphoid leukemia can be subcategorized based on the cell type and the number of cells in circulation [3]. CLL can arise from B-, T-, or natural killer (NK) cell clones. B-cell CLL (B-CLL) is the most common in humans, accounting for 95% of cases. However, in dogs, T-cell CLL (T-CLL) is more prevalent, with approximately 73% of canine CLL cases involving cluster of differentiation 3+ (CD3+) T lymphocyte proliferation. Additionally, 54% of dogs with CLL exhibit a large granular lymphocyte (LGL) morphology. B-CLL is less common (26%) compare with T-CLL [4]. B-CLL is a malignancy of small-sized B-cells in the blood and bone marrow [5]. By contrast, LGL T-CLL primarily arises in the spleen and bone marrow [4]. NK-CLL is extremely rare in dogs, and the only reported case is not associated with LGL morphology [6]. Furthermore, specific canine NK cell markers have not yet been established, and no reports have definitively identified NK-CLL.

Accurate diagnosis and classification of hematopoietic neoplasia, including CLL, require immunophenotyping through flow cytometry [7]. Additionally, Polymerase Chain Reaction for Antigen Receptor Rearrangements (PARR) is a valuable tool for distinguishing neoplastic lymphoid cells from inflammatory ones. The PARR assay targets the hypervariable regions of the T-cell receptor gamma and immunoglobulin heavy chain genes, helping to identify monoclonal expansions indicative of neoplasia, as opposed to the polyclonal nature of inflammatory lymphoid cells [8, 9].

Although CLL often follows an indolent course, chemotherapy may be indicated in cases of severe lymphocytosis, progressive disease with cytopenia, lymphadenomegaly, splenomegaly, fever, or infection [10].

Herein, we report a case of canine B-cell chronic lymphoid leukemia with a CD21^−^ phenotype using the LT21 clone CD21 antibody in flow cytometry.

## Case presentation

**Fig. 1:**
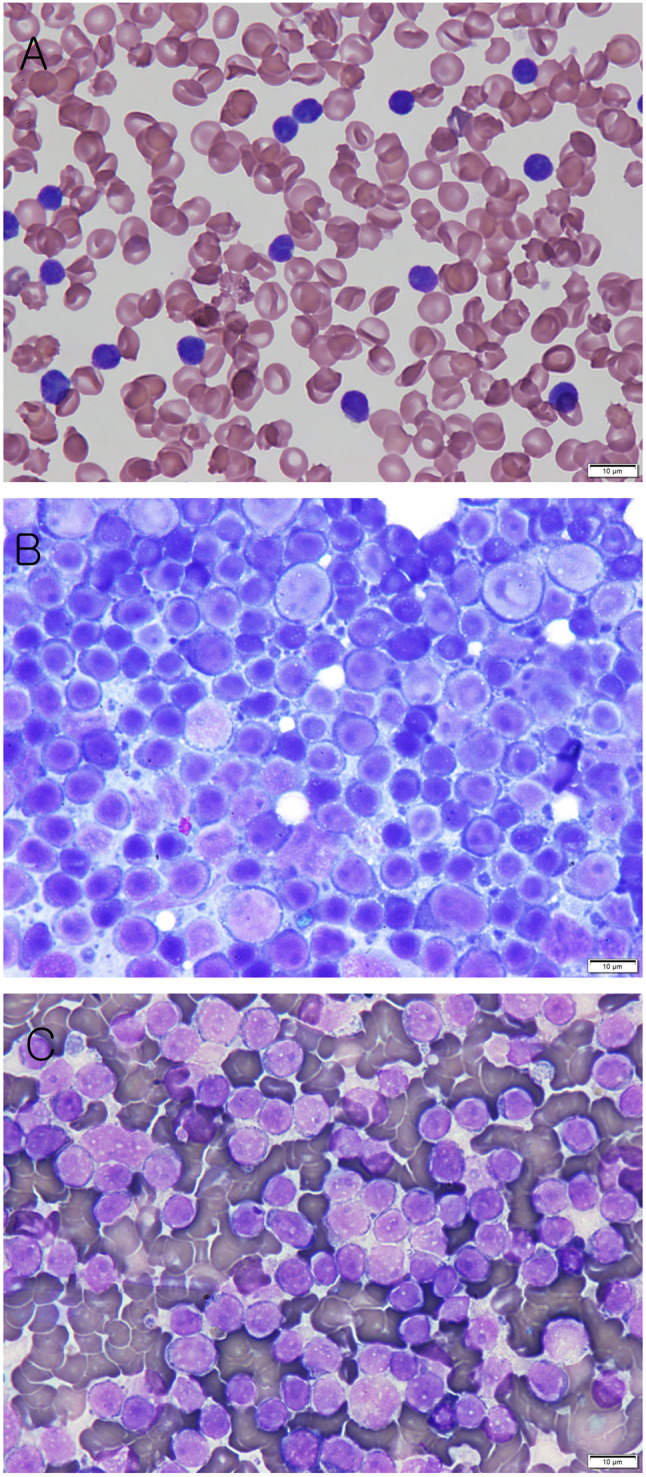
Cytological examination of the blood smear, inguinal lymph node and spleen. (**A**) Peripheral blood smear (Diff-quick stain, × 1,000, scale bar: 10 μm). (**B**) Fine-needle aspiration of the inguinal lymph node (Diff-quick stain, × 1000, scale bar: 10 μm). (**C**) Fine-needle aspiration of the spleen (Diff-quick stain, × 1000, scale bar: 10 μm)

A 15-year-old, 4.4-kg spayed female Shih Tzu was presented to the veterinary medical teaching hospital of Kangwon National University. Despite having a normal appetite and activity level, cervical and inguinal lymph node enlargement was noted on physical examination. A complete blood count (CBC) revealed severe leukocytosis (white blood cells: 171,240/µL, reference interval [RI]: 5,000–16,700/µL), severe lymphocytosis (lymphocyte: 163,540/µL, RI: 1,000–5,000/µL), and monocytosis (monocyte: 3,010/µL, RI: 160–1,120/µL). The red blood cell (RBC) and platelet counts were within the reference range (RBC: 5.68 M/µL, RI: 5.6–8.8 M/µL; platelet: 320 K/µL, RI: 148–484 K/µL). A serum biochemistry profile showed increased symmetric dimethylarginine (SDMA: 18 µg/dL; RI: 0–14 µg/dL). A differential leukocyte count was performed on blood smears, which yielded the following results: segmented neutrophils, 2%; small lymphocytes, 95%; large lymphocytes (lymphoblasts), 2%; and monocytes, 1%. Many small lymphocytes were observed, and severe lymphocytosis was confirmed (Fig. 1A). The corrected cell counts were as follows: neutrophil, 3,425/µL; lymphocyte, 166,102/µL; and monocyte, 1,712/µL. Splenomegaly, hepatomegaly, and lymph node enlargement were detected on radiographic and ultrasonographic examination. Cytological examination of the inguinal lymph node and spleen showed a predominance of small lymphocytes (Fig. 1B and C). The detailed cytology findings of the lymph node, as provided by a Diplomate of the American College of Veterinary Pathologists, are as follows. A differential cell count of 100 lymphoid cells performed on one of the slides yielded 73% small lymphocytes that had a rounded nucleus with condensed chromatin and a small volume of pale basophilic cytoplasm, 18% intermediate-sized lymphocytes that had a rounded nucleus with stippled chromatin and a small rim of pale basophilic cytoplasm, and 6% large lymphocytes that had a rounded nucleus with stippled chromatin, and a small rim of variably light to dark blue cytoplasm. There were also 3% plasma cells. A relatively low number of neutrophils were present, and the background contained red blood cells and free nuclei. Thus, the interpretation is consistent with a reactive/hyperplastic lymph node. The SNAP 4Dx Plus test was negative for heartworm, *Ehrlichia* spp., *Borrelia burgdorferi*, and *Anaplasma* spp. Two days later, the CBC test before chemotherapy showed mild anemia (RBC: 5.06 M/µL, RI: 5.6–8.8 M/µL; Hematocrit: 34.1%, RI: 37.3–61.7%; Hemoglobin: 11.7 g/dL, RI: 13.1–20.5 g/dL).

Peripheral blood mononuclear cells (PBMCs) were isolated from heparin-treated blood following a density gradient technique using Ficoll-Paque^®^ PLUS (density: 1.077 g/mL, GE Healthcare, Chicago, Illinois, USA). Immunophenotyping was conducted using various antibodies and isotype control antibodies in peripheral blood mononuclear cells (PBMCs) according to the manufacturer’s instructions (Table 1). The methods used for staining and the instruments used for analysis are described in the Supplementary Information. The amount of antibody used per well is listed in Table 2.

**Table 1:** Antibodies used for flow cytometry

Antibody (Isotype)	Clone	Supplier	Catalog number
Mouse anti-dog CD3-FITC (mouse IgG1)	CA17.2A12	Bio-Rad	MCA1774F
Mouse anti-dog CD21-FITC (Mouse IgG1)	LT21	Invitrogen	MA119753
Mouse anti-dog CD21-Alexa Fluor^®^ 647 (Mouse IgG1)	CA2.1D6	Bio-Rad	MCA1781A647
CD4/CD8 antibody cocktail,-FITC/ PE (Rat IgG1/IgG2a)	YKIX 302.9/ YCATE 55.9	Invitrogen	MA516990
Mouse anti-canine CD34-PE (Mouse IgG1)	1H6	ebioscience	12–0340
Rat anti-canine CD45-FITC (Rat IgG2b, κ)	YKIX716.13	ebioscience	11-5450
Rat anti-canine major histocompatibility complex (MHC) class II-FITC (Rat IgG2a, κ)	YKIX334.2	ebioscience	11-5909
Mouse anti-human CD14-FITC (Mouse IgG2a, κ)	M5E2 (RUO)	BD Bioscience	557,153
anti-dog CD5-Pacific Blue™	YKIX322.3	Bio-Rad	MCA1037PB
Mouse anti-dog CD94-Alexa Fluor^®^ 647 (Mouse IgG1)	8H10	Bio-Rad	MCA6400A647
**Intracellular staining**	**Clone**	**Supplier**	**Catalog number**
CD79a monoclonal antibody-PE (Mouse IgG1, κ)	HM47	Invitrogen	12-0792-42
Mouse anti-human Granzyme B-PE (Mouse BALB/c IgG1, κ)	GB11 (RUO)	BD Bioscience	561,142
**Isotype control**	**Clone**	**Supplier**	**Catalog number**
Mouse IgG1, κ-FITC (for CD3 and CD21)	MOPC-21	BD Bioscience	555,748
Mouse IgG1 negative control: Alexa Fluor^®^ 647	No search	Bio-Rad	MCA928A647
Mouse IgG1, κ-PE (for CD34, CD79a and Granzyme B)	MOPC-21 (RUO)	BD Bioscience	555,749
Rat IgG2b, κ-FITC (for CD45)	A95-1	BD Bioscience	556,923
Rat IgG2a, κ-FITC (for MHC class II)	R35-95	BD Bioscience	554,688
Mouse IgG2a,κ-FITC (for CD14)	G155-178 (RUO)	BD Bioscience	555,573
RAT IgG2a Negative Control-Pacific Blue^®^ (For CD5)	YTH71.3	Bio-Rad	MCA1212PB
Mouse IgG1 Negative Control-Alexa Fluor^®^ 647 (For CD94)	No search	Bio-Rad	MCA928A647
**Analysis combination**			
CD3/CD79a			
CD4/CD8			
CD21/CD79a			
CD3/CD5/CD94			
CD3/Granzyme B			
Other antibodies: single staining			

FITC: Fluorescein isothiocyanate; PE: phycoerythrin; Alexa 647: Alexa Fluor^®^ 647

**Table 2:** Amounts of antibodies used for flow cytometry analysis

Antibody	Amount of antibody (per well)
Mouse anti-dog CD3-FITC	A 10-fold dilution was prepared, and 10 µL was used.
Mouse anti-dog CD21-FITC Mouse anti-dog CD21-Alexa 647	20 µL A 10-fold dilution was prepared, and 10 µL was used
CD4/CD8 antibody cocktail,-FITC/ PE	10 µL
Mouse anti-canine CD34-PE	5 µL
Rat anti-canine CD45-FITC	5 µL
Rat anti-canine MHC class II-FITC	5 µL
Mouse anti-human CD14-FITC	20 µL
anti-dog CD5-Pacific Blue™	A 10-fold dilution was prepared, and 10 µL was used.
Mouse anti-dog CD94-Alexa	A 10-fold dilution was prepared, and 10 µL was used.
**Intracellular staining**	**Amount of antibody (per well)**
CD79a monoclonal antibody-PE	5 µL
Mouse anti-human Granzyme B-PE	5 µL
**Isotype control**	**Amount of antibody (per well)**
Mouse IgG1, κ-FITC (for CD3 and CD21)	5 µL
Mouse IgG1 negative control: Alexa Fluor^®^ 647	A 10-fold dilution was prepared, and 10 µL was used.
Mouse IgG1, κ-PE (for CD34, CD79a and Granzyme B)	5 µL
Rat IgG2b, κ-FITC (for CD45)	5 µL
Rat IgG2a, κ-FITC (for MHC class II)	5 µL
mouse IgG2a,κ-FITC (for CD14)	5 µL
RAT IgG2a Negative Control-Pacific Blue^®^ (For CD5)	A 10-fold dilution was prepared, and 10 µL was used.
Mouse IgG1 Negative Control-Alexa Fluor^®^ 647 (For CD94)	A 10-fold dilution was prepared, and 10 µL was used.

FITC: Fluorescein isothiocyanate; MHC: major histocompatibility complex; PE: phycoerythrin; Alexa 647: Alexa Fluor^®^ 647

**Fig. 2:**
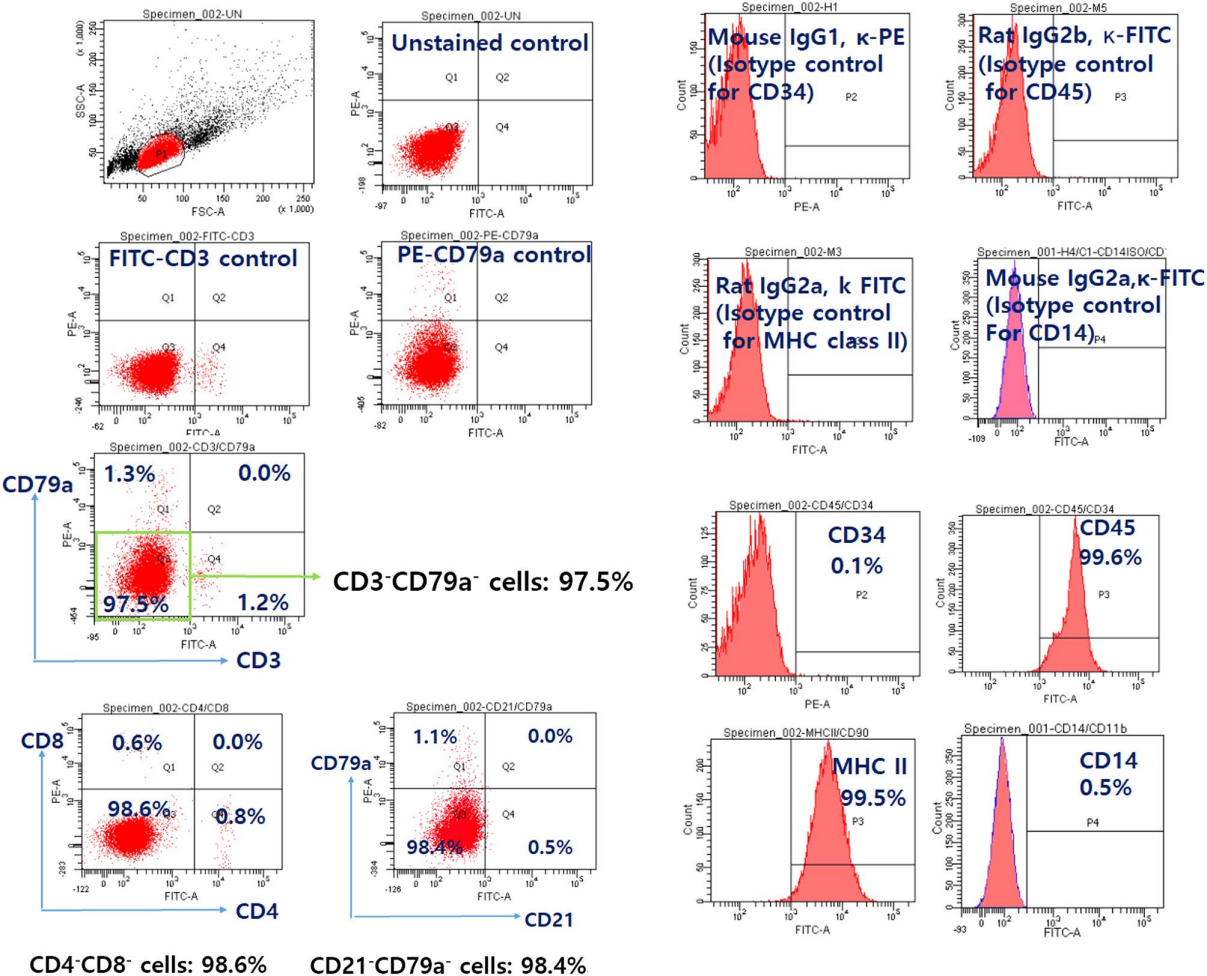
Immunophenotyping of peripheral mononuclear cells before chemotherapy. Peripheral blood mononuclear cells isolated from the canine patient were immunophenotyped by flow cytometric analysis using FITC-conjugated mouse anti-dog CD3/PE-conjugated mouse anti-CD79A, FITC-conjugated rat anti-dog CD4/PE-conjugated rat anti-dog CD8 monoclonal antibody, FITC-conjugated mouse anti-dog CD21/PE-conjugated mouse anti-CD79A, PE-conjugated mouse anti-canine CD34, FITC-conjugated rat ant-canine CD45, FITC-conjugated rat anti-canine MHC class II, and FITC-conjugated mouse anti-human CD14 antibodies. Nonspecific binding of targeted antibodies to cell surface antigens was estimated using isotype control antibodies. Gating was performed with reference to populations of unstained, single stained, and isotype controls. FITC: fluorescein isothiocyanate; PE: phycoerythrin

The majority of lymphocytes from this patient showed CD3^−^CD79a^−^ (97.5%), CD4^−^CD8^−^ (98.6%), CD21^−^CD79a^−^ (98.4%), CD34^−^ (0.1%), CD45^+^ (99.6%), major histocompatibility complex (MHC) class II ^+^ (99.5%), and CD14^−^ cells (0.5%) (Fig. 2). Based on the immunophenotyping results, possible differentials considered include the following: the majority of lymphocytes may be NK cell clones, plasma cell clones, or show aberrant expression or loss of CD21 marker due to the neoplastic nature of the cells.

**Fig. 3:**
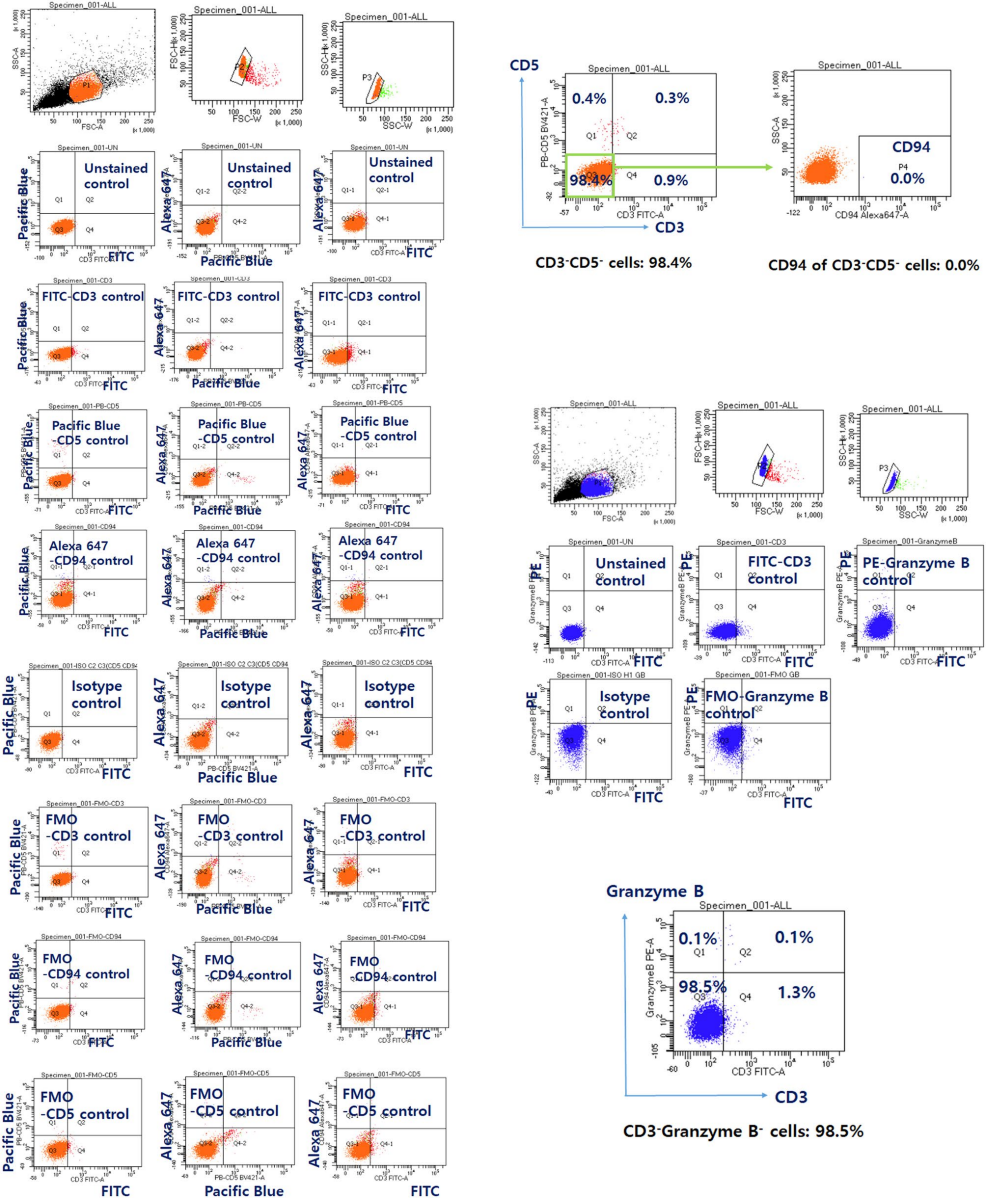
Additional immunophenotyping of peripheral mononuclear cells. Peripheral mononuclear cells isolated from the canine patient were immunophenotyped by flow cytometric analysis using FITC-conjugated mouse anti-dog CD3/Pacific Blue™-conjugated anti-dog CD5/Alexa Fluor^®^ 647-conjugated mouse anti-dog CD94, and FITC-conjugated mouse anti-dog CD3/PE-conjugated mouse anti-human Granzyme B. The nonspecific binding of targeted antibodies to cell surface antigens was estimated using isotype control antibodies. Gating was performed with reference to populations of unstained, single stained, isotype, and FMO controls. FITC: fluorescein isothiocyanate; PE: phycoerythrin; FMO: fluorescence minus one

Before staining with granzyme B, an antibody requiring intracellular staining, the BD Cytofix/Cytoperm™ Fixation/Permeabilization Kit was used according to the manufacturer’s instructions. Subsequently, additional flow cytometry was performed using antibodies against CD3, CD5, CD94, and granzyme B (Fig. 3). Upon combining the flow cytometry results, the majority of lymphocytes in this patient’s PBMC was determined to exhibit the cell subset of CD3^−^CD5^−^CD21^−^ CD94^−^granzyme B^−^ phenotype.

**Fig. 4:**
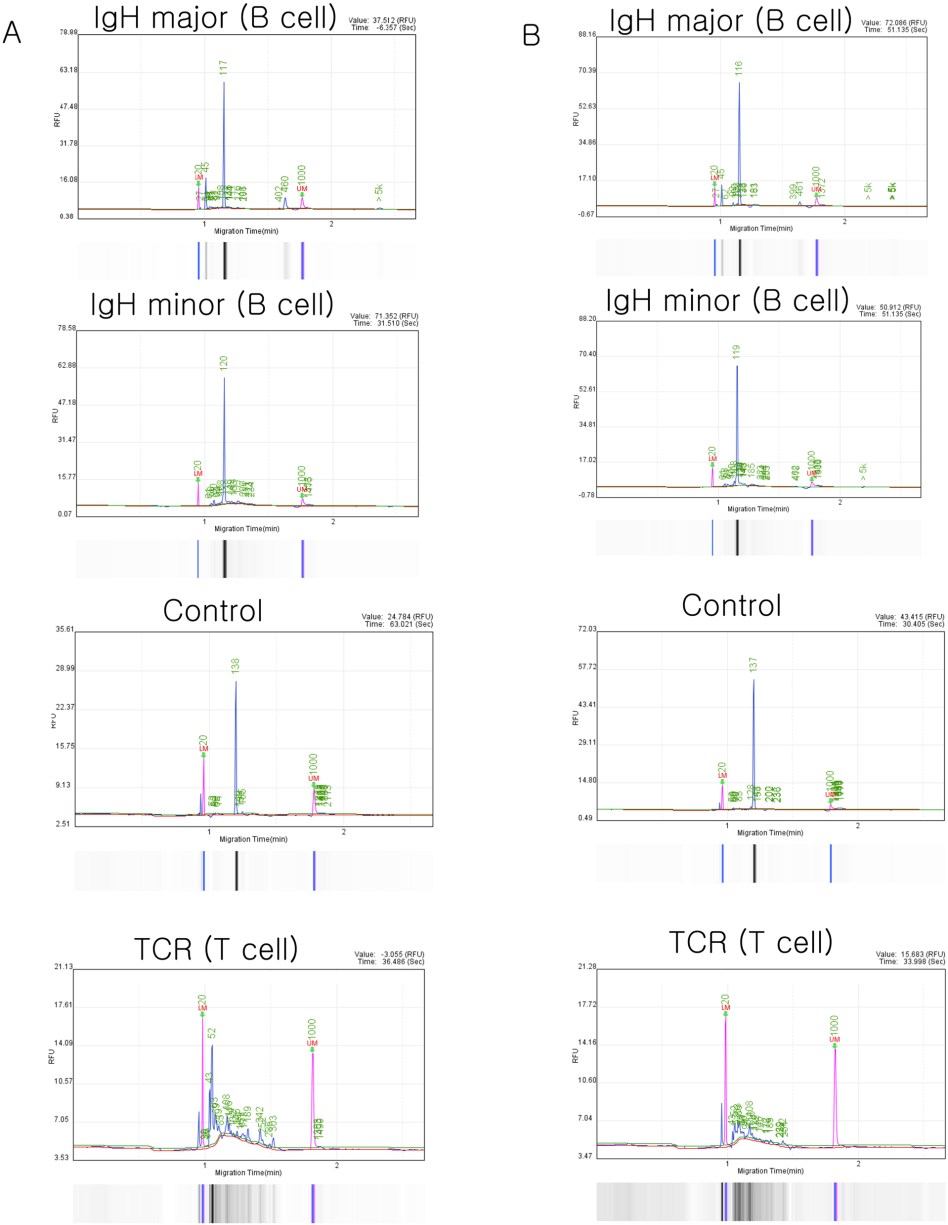
Capillary electrophoresis traces of PARR using DNA samples from a blood smear slide and an inguinal lymph node slide provided by the reference laboratory. Both (**A**) blood and (**B**) lymph node samples showed monoclonality in IgH major and IgH minor. PARR performed using IgH major (target Tm: 85ºC and target bp: 120), IgH minor (target Tm: 86ºC and target bp: 120), TCR (target Tm: 83ºC, target bp: 90), and lymphocyte control primers (target bp: 130). bp, base pair; Tm, melting temperature; PARR, polymerase chain reaction for antigen receptor rearrangement; IgH, immunoglobulin heavy chain; TCR, T-cell antigen receptor

To confirm monoclonality and exclude the aberrant loss of CD21 marker, a blood smear slide and an inguinal lymph node slide obtained from the first visit were sent to the reference laboratory (GREEN VET, Yongin, South Korea) for PARR testing. The primers and PCR protocol used for PARR have been described previously [11]. PARR tests of blood smears and inguinal lymph node slides showed B-cell monoclonality (Fig. 4A and B). The polyclonal B-cell control for PARR is shown in Supplementary Fig. 3.

To rule out or rule in plasma cell origin, immunocytochemistry was performed using the primary antibody against Multiple Myeloma Oncogene 1 (MUM1, 1:400, Biocare Medical, CRM352B). As secondary antibody, Alexa 488-goat anti-rabbit IgG (10 µg/mL, Invitrogen, A11034) was used, respectively. The detailed staining methods for immunocytochemistry are described in the Supplementary Information. Immunocytochemistry using PBMCs showed that the plasma cell marker MUM1 was not expressed (Supplementary Fig. 4). Therefore, the diagnosis was confirmed to be CD21 negative B-CLL.

On day 2, after the diagnosis of CLL, the patient was treated with melphalan (Alkeran, Excella GmbH & Co. KG, Feucht, Germany) 3 mg/m² once a day (semel in die, SID) and prednisolone (Solondo Tab., Yuhan Corporation, Seoul, South Korea) 2 mg/kg SID per os (PO). Prednisolone dosage was reduced by 25% each week. Based on the white blood cell (WBC) and lymphocyte counts from the CBC test, if remission was achieved, the chemotherapy was discontinued. If a relapse occurred, the treatment was resumed. On day 17, the WBC count returned to 7,650/µL, within the reference range, and the lymphocyte count was 670/µL. Furthermore, both lymphadenopathy and splenomegaly were reduced. Thus, melphalan was discontinued, and a tapering dose of prednisolone was continued until discontinuation. Lymphadenopathy resolved two weeks after the WBC count returned to the normal reference range (day 31). However, on day 103, lymphocytosis (33,640/µL) was confirmed during a routine health check. The duration of the first remission period was 87 days. Thus, the same chemotherapy regimen was reintroduced to the patient (melphalan 3 mg/m² SID PO; prednisolone 2 mg/kg SID PO with 25% dose-tapering every week). After 10 days, the lymphocyte count was 1,200/µL; however, hematocrit (HCT) values were below the normal reference range (day 113). Thus, melphalan was discontinued again, and the prednisolone dosage was tapered as before. On day 198, the patient’s lymphocyte count was 11,150/µL, and no other abnormalities were observed. The duration of the second remission period was 85 days. Since there were no clinical signs and no other hematological abnormalities, the administration of chemotherapeutic agents was delayed. However, on day 277, a severely elevated lymphocyte count (55,170/µL) with mild non-regenerative anemia (HCT 35.9%) was noted. The patient was treated with melphalan 5 mg/m² SID PO and prednisolone 2 mg/kg SID PO with 25% dose-tapering every week. After 17 days (day 294), the lymphocyte count returned to the normal reference range (2,319/µL). On day 386, a routine CBC test result revealed lymphocytosis (14,180/µL) without any abnormalities. The remission duration was 92 days. On day 447, splenomegaly and lymphadenopathy were observed on diagnostic imaging with severe lymphocytosis (75,720/µL) and mild non-regenerative anemia (HCT 36.7%). Considering the resistance observed to previous treatments, melphalan was replaced with Chlorambucil (Leukeran, Excella GmbH & Co. KG, Feucht, Germany) at 3 mg/m² SID PO for four weeks and prednisolone at 2 mg/kg SID PO with 25% dose-reduction every week. However, the lymphocyte count did not return to the normal reference range; thus, Chlorambucil dosage was increased to 6 mg/m² SID PO for another four weeks with prednisolone at 2 mg/kg SID PO, with 25% dose-reduction every week. This dose escalation resulted in the lymphocyte count remaining within the reference range for 88 days. On day 591, the patient showed mild lymphocytosis without other abnormalities, and on day 704, the lymphocyte counts were markedly elevated (197,460/µL); thus, the previous treatment regimen was reintroduced. However, complete remission was not achieved, and the patient no longer responded to Chlorambucil nor to other treatments such as Imatinib (Gleevec, Novartis Pharma Stein AG, Stein, Switzerland) and cyclophosphamide of CHOP protocol. On day 824, further treatment was declined by the owner, and the patient died at home after 60 days. The patient survived for 884 days after the diagnosis of CLL.

**Fig. 5:**
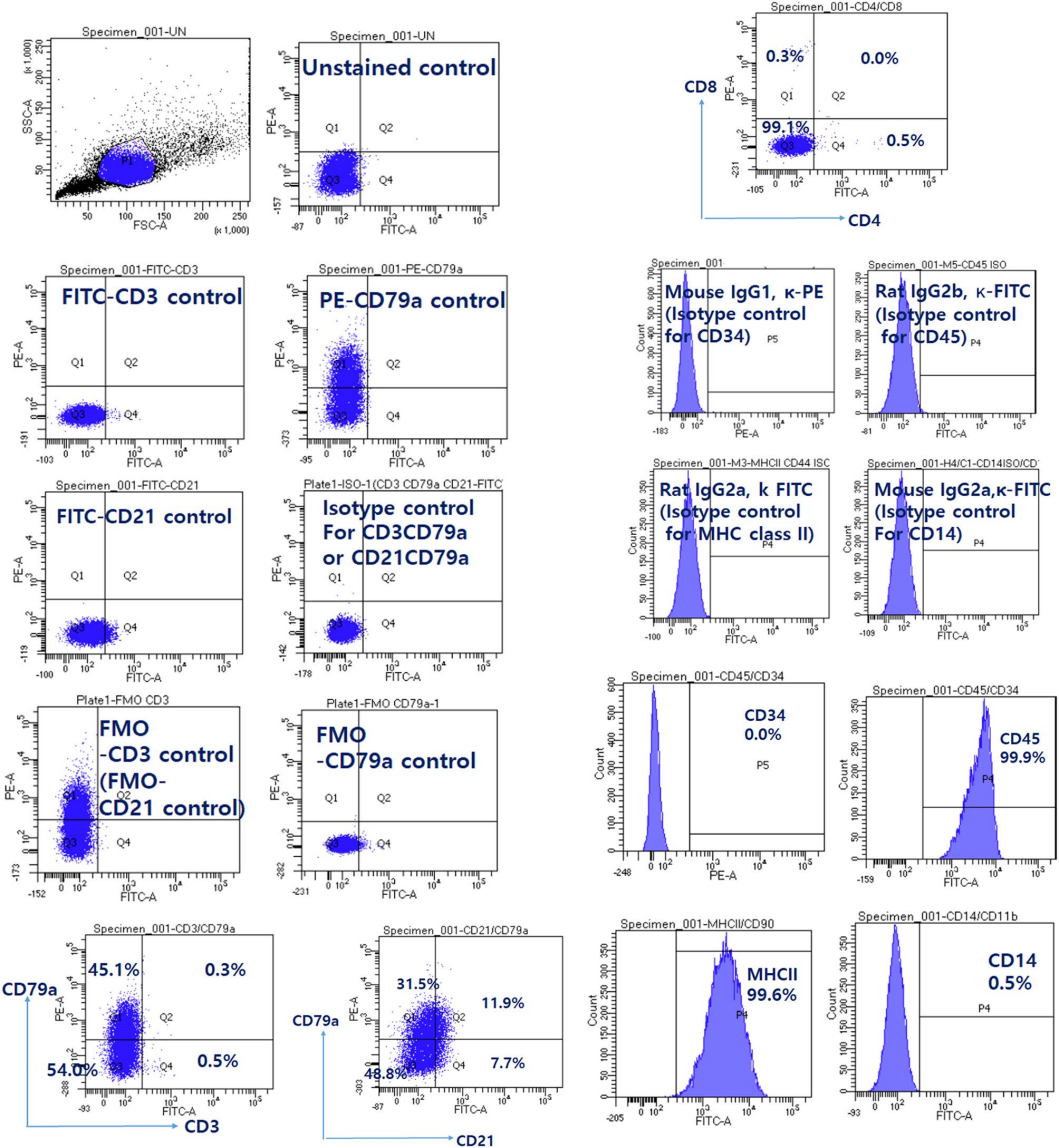
Immunophenotyping of peripheral mononuclear cells performed at day 793 after chronic lymphoid leukemia diagnosis. Peripheral mononuclear cells isolated from the canine patient were immunophenotyped by flow cytometric analysis using FITC-conjugated mouse anti-dog CD3/PE-conjugated mouse anti-CD79A, FITC-conjugated rat anti-dog CD4/PE-conjugated rat anti-dog CD8 monoclonal antibody, FITC-conjugated mouse anti-dog CD21/PE-conjugated mouse anti-CD79A, PE-conjugated mouse anti-canine CD34, FITC-conjugated rat ant-canine CD45, FITC-conjugated rat anti-canine MHC class II, and FITC-conjugated mouse anti-human CD14 antibodies. Nonspecific binding of targeted antibodies to cell surface antigens was estimated using isotype control antibodies. Gating was performed with reference to populations of unstained, single stained, isotype, and FMO controls

The FACS data of PBMCs collected on day 793 are presented in Fig. 5. The expression of CD21 (19.6%) and CD79a (45.4%) was observed in the FACS results after chemotherapy. The aberrant phenotype was altered, which could indicate that this patient was in clinical remission (Fig. 5). When comparing CD21 expression on the patient’s PBMCs preserved in liquid nitrogen using the clone LT21 and the newly purchased clone CA2.1D6 CD21 antibodies, strong expression was observed with the clone CA2.1D6 CD21 antibody (Supplementary Fig. 5).

### Discussion and conclusions

Canine CLL is a slow-progressing and often indolent disease. In the present case, the patient was asymptomatic and incidentally discovered during routine CBC tests.

Immunophenotyping is widely used to precisely diagnose and classify various types of hematopoietic neoplasia [7, 12]. CD3 is expressed by T cells, CD79a is expressed by immature and mature B cells and in most types of B-cell neoplasms, and CD21 is expressed by mature B cells [13].

In this study, the majority of lymphocytes from this patient showed CD3^−^CD79a^−^ (97.5%), CD4^−^CD8^−^ (98.6%), CD21^−^CD79a^−^ (98.4%), CD34^−^ (0.1%), CD45^+^ (99.6%), and MHC class II^+^ cells (99.5%). The majority of lymphocytes may be NK cell clones, plasma cell clone or show aberrant expression or loss of CD markers due to its neoplastic nature.

In dogs, if persistent small-cell lymphocytosis shows a homogeneous population of lymphocytes in which more than 80% of the lymphocytes share the same immunophenotype, this suggests neoplastic lymphocytosis [14].

Almost all human and canine CLL have been reported as CD34^−^, while acute lymphoblastic leukemia (ALL) may exhibit concurrent CD34 expression; therefore, CD34 expression serves as a useful marker for distinguishing CLL from ALL [4]. CD34 expression is commonly observed in acute, non-LGL leukemia of both myeloid and lymphoid origins in dogs [4].

NK cells are key components of the body’s innate immune system, serving as its primary early defense mechanism. They selectively kill cells without prior activation [15]. NK cells recognize malignant cells and respond rapidly without prior sensitization. Activated NK cells release cytotoxic granules containing granzymes and perforin to lyse tumor cells [15]. According to a previous study that isolated and characterized normal canine NK cells, NK cells were identified as non-T and non-B cells (CD3 or CD5: 0.94 ± 1%, CD4: 0.50 ± 0.9%, and CD22: 1.3 ± 1%). Additionally, NK cells were not of monocyte origin (CD14: 0.16 ± 0.28%). They universally (> 95%) expressed CD45 and MHC class I. The expression levels were 79.6 ± 3.6% for MHC class II, 52 ± 34% for CD11/18, and 42 ± 30% for CD11b [12]. Furthermore, canine NK cells did not bind anti-human CD56 or anti-human NKp46 antibodies [12]. Prototypic murine or human NK cell markers, including NK1.1, CD56, Ly49, and KIR, are not available as canine NK markers [16–19].

Specific canine NK cell markers have not yet been established. However, CD3^−^CD5^−^CD21^−^ was identified as NK lymphocytes in Lee et al.’s study [20], while CD5^−^CD21^−^ cells demonstrated NK activity in Lin et al.’s study [21].

When additional flow cytometry on PBMC was performed using antibodies against CD3, CD5, CD94, and granzyme B, combined flow cytometry results showed that the majority of lymphocytes of this patient was CD3^−^CD5^−^CD21^−^, with no expression of granzyme B and CD94. The absence of Granzyme B and CD94 expression in these cells reduces the likelihood that the leukemia is of NK cell origin.

To confirm monoclonality and exclude the aberrant loss of CD markers, PARR tests using blood smear slides and inguinal lymph node slides obtained at the first visit revealed B-cell monoclonality.

Plasma cell leukemia and B cell acute lymphoblastic leukemia may also show B cell monoclonality, but considering the cell shape and size in cytology, it could be excluded. Plasma cells are characterized by eccentric nuclei, basophilic cytoplasm, and a perinuclear clear zone (Golgi apparatus). Furthermore, in acute lymphoblastic leukemia, tumor cells appear in blood as lymphoblasts. In a case report of a dog with plasma cell leukemia, immunohistochemistry revealed that the tumor cells were negative for CD204, IBA-1, E-cadherin, CD3, CD5, CD79a, CD20, and PAX5 but positive for MUM1. Therefore, IRF4/MUM1 expression should be included in additional tests to differentiate plasma cell leukemia [22]. In immunocytochemistry using PBMCs, the plasma cell marker MUM1 was not expressed. Therefore, it is highly unlikely that this patient has plasma cell leukemia. ALL primarily involves the bone marrow, which is hypercellular and usually replaced by an overabundance of lymphoblasts. Clinical signs are typically acute at onset, due to the infiltrative and functional effects of the expanding burden of malignant cells, and are most commonly a consequence of disrupted hematopoiesis [23].

The fact that the main site of involvement is the bone marrow and blood helps distinguish CLL from small-cell lymphoma stage V. However, the distinction between CLL and small-cell lymphoma stage V is challenging [1]. Lymphadenopathy in CLL is either absent or moderate, but it is characterized by drastic enlargement in small-cell lymphoma. In this case, the main site of involvement was the blood, and the patient exhibited mild anemia and mild lymphadenopathy. Moreover, small-cell lymphoma stage V with lymphocyte count > 160,000/µL was not reported. Therefore, a diagnosis of CLL was made.

The PARR assay is a widely used method for diagnosing lymphoproliferative neoplasia in cases involving enlarged lymph nodes, organs, and bone marrow samples. Nevertheless, clonal, “false positive” results from disorders other than lymphatic neoplasia can occur. These conditions include some infectious diseases [24, 25], myeloproliferative diseases [26], and atypical lymphoid hyperplasia in humans [27]. This topic has been discussed in detail by Melendez-Lazo et al. [28].

In this study, an aberrant immunophenotype of CD21^−^ was observed in a dog with B-CLL. Previous studies on the aberrant immunophenotypes of neoplastic lymphoid cells have been reported in dogs [29–31]. Gelain et al. reported that diminished CD79a expression was observed in 45% of B-cell lymphomas [29]. According to a recent report on aberrant immunophenotypes in canine lymphoma, 24% (4/17) of the B-cell lymphomas examined were CD79a^−^, and 6% (1/17) were CD3^−^/CD21^−^ [32].

According to a previous study, anemia occurs in 58% of dogs with CLL, while thrombocytopenia is observed in 27% of cases [4]. In this patient, RBC and platelet counts were within reference ranges, but two days later, the CBC test prior to chemotherapy showed mild anemia. Monoclonal gammopathy-related hyperproteinemia can occur in dogs with B-cell CLL, but was not observed in this case. The dog presented with severe lymphocytosis, hepatomegaly, and splenomegaly, prompting the initiation of chemotherapy. In our patient, the aberrant phenotype was altered after chemotherapy, which could indicate that this patient was in clinical remission.

We purchased a different clone of CD21 (clone CA2.1D6), which showed strong expression of CD21 in this patient’s PBMCs stored in liquid nitrogen, indicating that the CD21 from the LT21 clone had a very weak reaction with the patient’s B cells (Supplementary Fig. 5). Since the initial sample was not preserved, we were unable to confirm the CD21 expression with the CA2.1D6 clone. Therefore, it is possible that the sample was genuinely CD21 negative, but it is also likely that the weak response of the LT21 clone resulted in a CD21 negative outcome in the flow cytometry analysis. Further study is needed to compare CD21 expression levels using Clone LT21 and Clone CA2.1D6 antibodies in a larger cohort of healthy dogs and canine patients.

In conclusion, this is the first report of canine B-CLL with CD 21 negativity. The neoplastic nature of B-CLL might lead to loss of the CD21 marker. Immunophenotyping can aid in subclassifying the type of lymphoid leukemia; however, due to the potential aberrant expression or loss of CD markers in tumor cells, a comprehensive assessment combining the PARR technique would provide more reliable results.

## Electronic supplementary material

Below is the link to the electronic supplementary material.

Supplementary Material 1

## Data Availability

All data generated or analyzed during this study are included in this published article.

## Abbreviations

ALL: Acute lymphoblastic leukemia
CLL: Chronic lymphoid leukemia
NK: Natural killer
CD: Cluster of differentiation
LGL: Large granular lymphocyte
CBC: Complete blood counts
WBC: White blood cell
RI: Reference interval
RBC: Red blood cell
SDMA: Symmetric dimethylarginine
PBMCs: Peripheral blood mononuclear cells
FITC: Fluorescein isothiocyanate
PE: Phycoerythrin
MHC: Major histocompatibility complex
PARR: Polymerase chain reaction for antigen receptor rearrangement
