# Supplementary material for: Diagnosis of canine B-cell chronic lymphoid leukemia with a CD21 negative phenotype using the LT21 clone CD21 antibody in flow cytometry: a case report
Source: BMC Vet Res. 2024 Oct 26;20:490. doi: 10.1186/s12917-024-04335-x (PMC11515120; doi:10.1186/s12917-024-04335-x)
Supplement: Supplementary file 1 — Supplementary Material 1 [file 12917_2024_4335_MOESM1_ESM.pdf]

Supplementary Information

Diagnosis of canine B-cell chronic lymphoid leukemia with a CD21 negative phenotype using the LT21 clone CD21 antibody in flow cytometry: a case report

Eun Wha Choi*, Yunho Jeong, Jin-Ok Ahn

[1]Department of Veterinary Clinical Pathology, College of Veterinary Medicine & Institute of Veterinary Science, Kangwon National University, 1 Kangwondaehak-gil, Chuncheon, Gangwon-do, 24341, Republic of Korea

[2]Department of Veterinary Internal Medicine, College of Veterinary Medicine, Kangwon National University, 1 Kangwondaehak-gil, Chuncheon, Gangwon-do, 24341, Republic of Korea

***Corresponding author:** Eun Wha Choi, DVM, PhD

Department of Veterinary Clinical Pathology, College of Veterinary Medicine & Institute of Veterinary Science, Kangwon National University, 1 Kangwondaehak-gil, Chuncheon, Gangwon-do, 24341, Republic of Korea

Phone: 82-33-250-8794

Fax: 82-33-259-5625

E-mail: ewchoi@kangwon.ac.kr

Staining method for immunophenotyping

For staining surface markers, approximately 10^6 cells in staining buffer (Dulbecco's phosphate-buffered saline/5% fetal bovine serum) were stained with the appropriate amount of fluorochrome-conjugated antibody specific to cell surface antigens at 4°C for 30 min in the dark (Total volume: 100 µL). The cells were then washed three times with 250 µL of staining buffer per well in microwell plates and pelleted by centrifugation at 500 × *g* for 5 min. The cells were resuspended in DPBS containing 2% PFA/2% FBS and stored at 4°C in the dark until flow cytometry analysis.

To stain both the surface and intracellular markers, the surface markers were stained, washed, and pelleted as described above. The cells are thoroughly resuspended, and 100 µL of Fixation/Permeabilization solution is added per well in microwell plates, followed by incubation for 20 min at 4°C. The cells are then washed two times in 1× BD Perm/Wash™ buffer (250 µL/well) and pelleted by centrifugation at 500 × *g* for 5 min. The fixed and permeabilized cells are thoroughly resuspended in 95 µL of BD Perm/Wash™ buffer containing an optimal concentration of fluorochrome-conjugated anti-CD79a or anti-Granzyme B antibody and incubated at 4°C for 30 min in the dark. The cells are then washed two times in 1× BD Perm/Wash™ buffer (250 µL/well) and pelleted by centrifugation at 500 × *g* for 5 min. Finally, the cells were resuspended in DPBS containing 2% PFA/2% FBS and stored at 4°C in the dark until flow cytometry analysis. Flow cytometry analysis was performed within 16 h using a FACSymphony A3 (Becton Dickinson, Franklin Lakes, NJ, USA), and the data were analyzed using BD FACSDiva Software (BD). The healthy control (CD21) is presented in Supplementary Figure 1, and the positive control (a dog patient with B-cell acute lymphoblastic leukemia) for CD79a is presented in Supplementary Figure 2.

A brief description of microcapillary electrophoresis for PARR assay

In Figure 4, the numbers on the peaks refer to the base pairs (bps) of that peak, and RFU represent the Relative Fluorescence Unit. This unit represents the intensity of the fluorescence signal measured using the fluorescence detection device. The RFU value is the relative expression of the fluorescence intensity emitted by a fluorescent dye bound to a specific molecule during analysis. ”Peak 20 bp” is the lower margin marker, “peak 1000 bp” is the upper margin marker, and the target peak (Target bp) is IgH major (target Tm: 85ºC and target bp: 120), IgH minor (target Tm: 86ºC and target bp: 120), and TCR (target Tm: 83ºC, target bp: 90). The positive control was used to verify the presence of lymphocytes within a given specimen (target bp: 130). The microcapillary electrophoresis equipment showed the base pair product between the lower margin marker and upper margin marker as a peak, whereas in the case of monoclonality, a very strong peak was generated at the target base pair.

Staining method for the immunocytochemistry

Peripheral blood mononuclear cells (PBMCs) were smeared onto silane-treated slides (MUTO PURE CHEMICALS Co. LTD, 5116-20F) and air-dried. The slide was fixed in methanol at −20 °C for 10 min and washed thrice with phosphate buffered saline (PBS, Sigma-Aldrich, Burlington, MA, USA) for 2 min. For blocking, the slides were incubated in 2% bovine serum albumin (BSA)/PBS at room temperature for 1 hour. The primary antibody against Multiple Myeloma Oncogene 1 (MUM1, 1:400, Biocare Medical, CRM352B) was diluted with 1% BSA/PBS (Komabiotech, Seoul, South Korea). Following overnight incubation with the primary antibody at 4 °C, the slide was rinsed thrice in PBS for 2 min. The secondary antibody

Alexa Fluor 488 goat anti-rabbit immunoglobulin G (IgG) (10 μg/ml, Invitrogen, A11034) was diluted with 1% BSA/PBS. Following overnight incubation with the secondary antibody at 4 °C, the slide was rinsed thrice in PBS for 2 min. The slides were then counterstained with mounting medium containing DAPI (Vector Laboratories, Burlingame, CA, USA) and examined using an LSM 880 laser-scanning confocal microscope (Carl Zeiss, Jena, Germany).

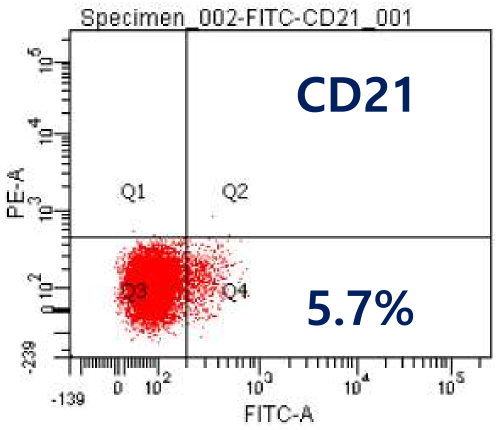

Supplementary Figure 1. Healthy control of CD21. Peripheral mononuclear cells isolated from the canine patient were immunophenotyped by flow cytometric analysis using FITC-conjugated mouse anti-dog CD21. In the healthy control (CD21), the population was divided into two categories based on the FITC gating axis of CD21 (10^{2}+1).

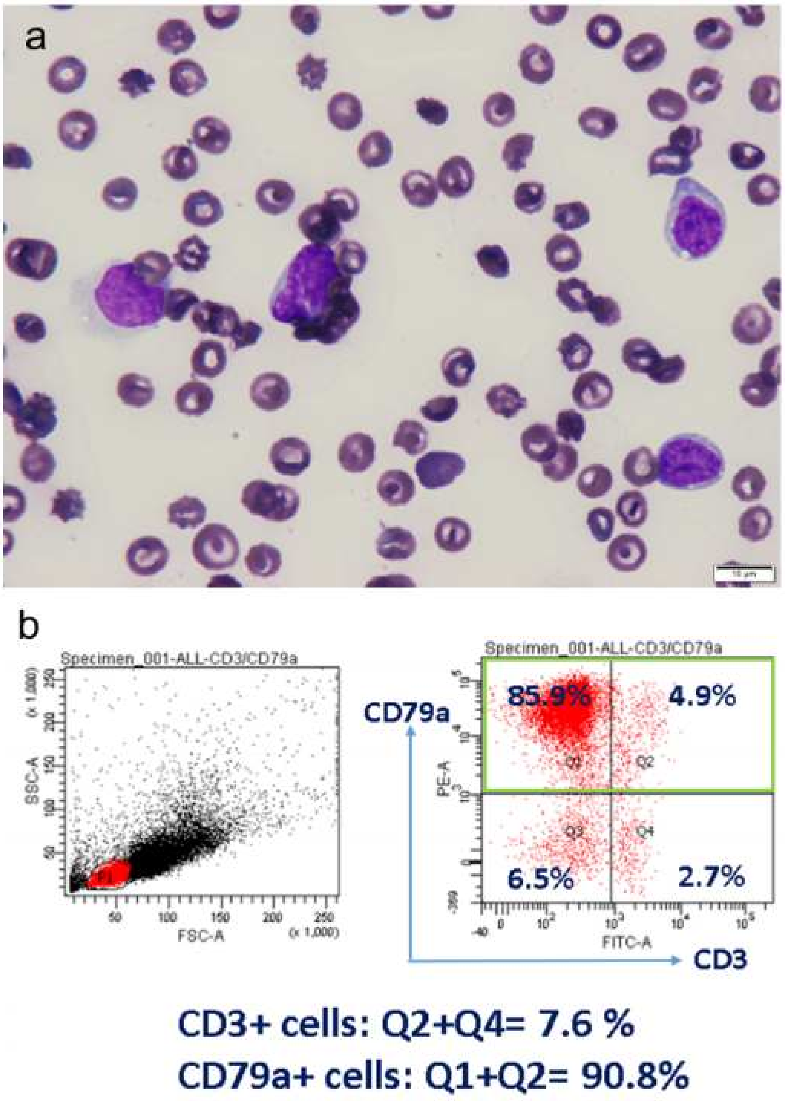

Supplementary Figure 2. Positive control of CD79a (a dog patient with B-cell acute lymphoblastic leukemia). (A) Peripheral blood smear (Diff-quick stain, × 1,000, scale bar: 10 μm). (B) Immunophenotyping of peripheral mononuclear cells. Peripheral mononuclear cells isolated from the canine patient were immunophenotyped by flow cytometric analysis using FITC-conjugated mouse anti-dog CD21/PE-conjugated mouse anti-CD79A

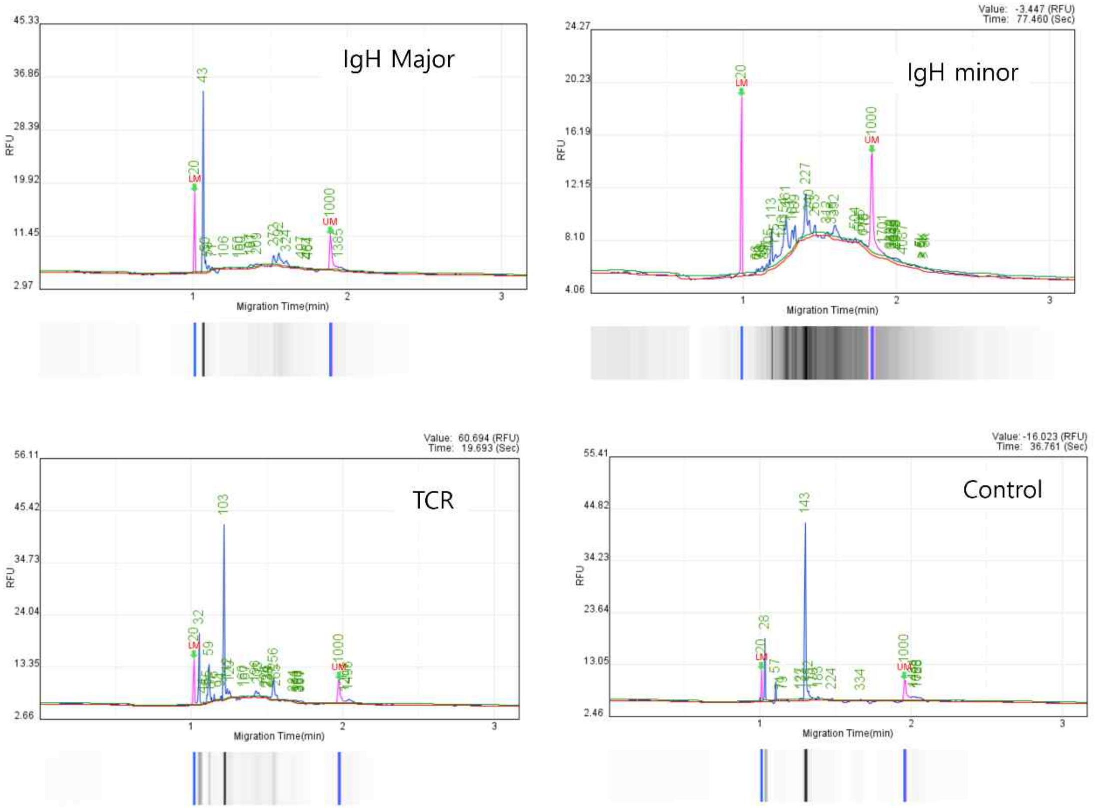

Supplementary Figure 3. Polyclonal B cell control (a dog patient with T cell lymphoma)-capillary electrophoresis traces of PARR using DNA samples from lymph node slide provided by the reference laboratory. PARR performed using IgH major (target Tm: 85ºC and target bp: 120), IgH minor (target Tm: 86ºC and target bp: 120), TCR (target Tm: 83ºC, target bp: 90), and lymphocyte control primers (target bp: 130). bp, base pair; Tm, melting temperature; PARR, polymerase chain reaction for antigen receptor rearrangement; IgH, immunoglobulin heavy chain; TCR, T-cell antigen receptor

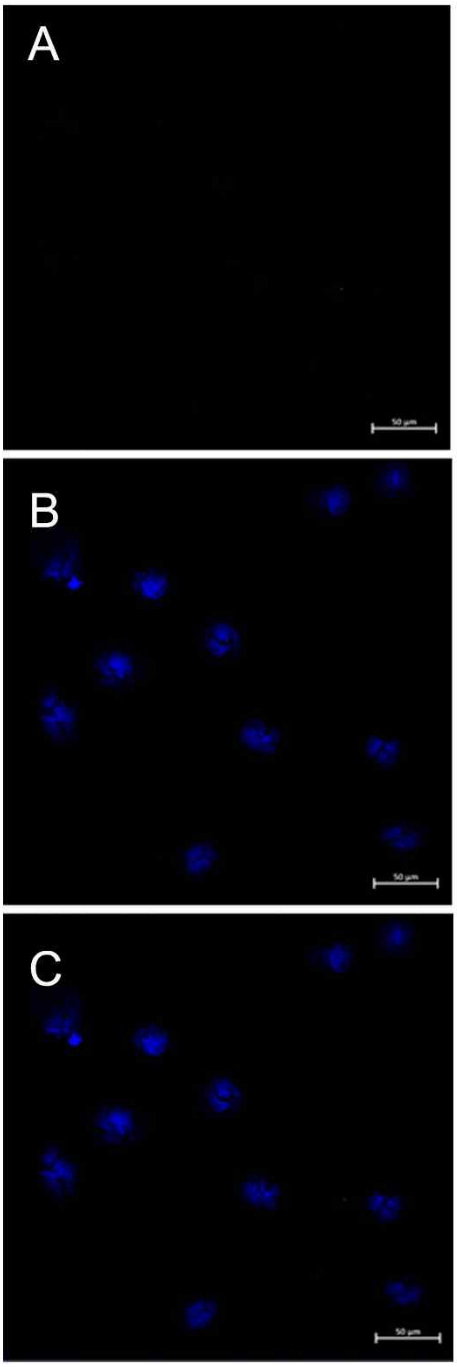

Supplementary Figure 4. Immunocytochemistry of peripheral mononuclear cells for Multiple Myeloma Oncogene 1 (MUM1). (A) MUM1 (green), (B) DAPI (Blue), (C) merged image of A and B (scale bar: 50 μm).

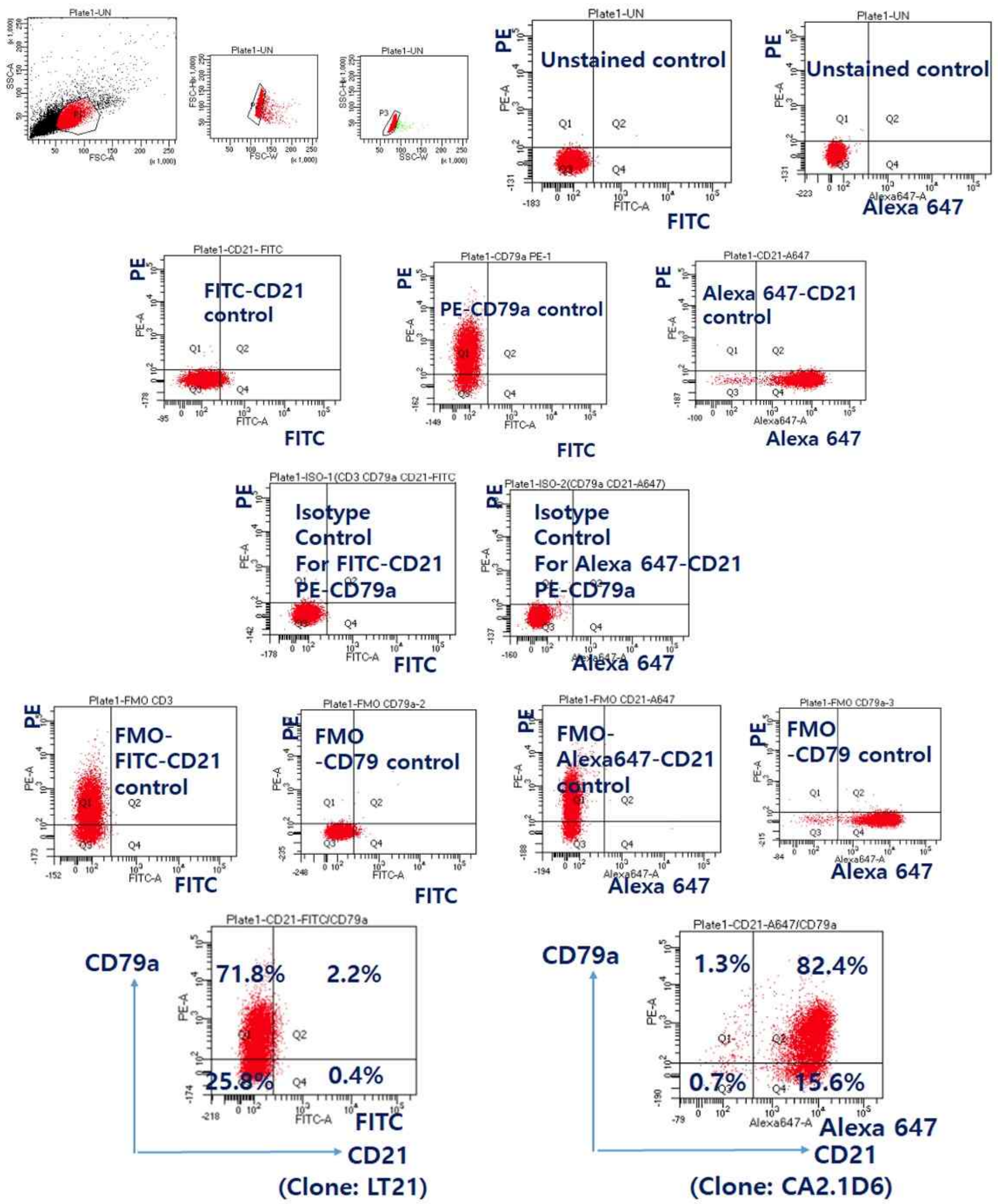

Supplementary Figure 5. Comparison of CD21 Expression Using Clone LT21 and Clone CA2.1D6 antibodies in patient PBMCs. Peripheral blood mononuclear cells isolated from the canine patient were immunophenotyped by flow cytometric analysis using FITC-conjugated mouse anti-dog CD21/PE-conjugated mouse anti-CD79A, and Alexa 647- mouse anti-dog CD21/ PE-conjugated mouse anti-CD79A. Gating was performed with reference to populations of unstained, single stained, and isotype controls. FITC: fluorescein isothiocyanate; PE: phycoerythrin; Alexa 647: Alexa Fluor® 647
